# Mr. Arthur Blair Avarne

**Published:** 1910-04-30

**Authors:** 


### OBITUARY.

The death is announced of Mr. Arthur Blair Avarne, M.R.C.S., L.S.A., of Blaenavon, Monmouth. Mr. Avarne, who died after a serious operation, became an L.S.A. in 1886, and two years later became a member of the Royal College of Surgeons.

